# Synergistic Effect of Cefazolin Plus Fosfomycin Against *Staphylococcus aureus in vitro* and *in vivo* in an Experimental *Galleria mellonella* Model

**DOI:** 10.3389/fphar.2021.685807

**Published:** 2021-05-11

**Authors:** Manuel Kussmann, Markus Obermueller, Matthias Karer, Richard Kriz, Rui-Yang Chen, Lena Hohl, Lisa Schneider, Heinz Burgmann, Ludwig Traby, Matthias G. Vossen

**Affiliations:** ^1^ Department of Medicine I, Division of Infectious Diseases and Tropical Medicine, Medical University of Vienna, Vienna, Austria; ^2^ Department of Clinical Pharmacology, Medical University Vienna, Vienna, Austria

**Keywords:** methicillin-resistant *Staphylococcus aureus*, synergy, combination therapy, antibiotic resistance, narrow-spectrum beta-lactam, rescue therapy

## Abstract

**Objectives:** This study investigated the synergistic *in vitro* and *in vivo* activity of cefazolin plus fosfomycin against methicillin-susceptible and methicillin-resistant *S. aureus* (MSSA and MRSA) to provide the basis for a potential treatment alternative.

**Methods:** Antimicrobial susceptibility and *in vitro* synergy tests were performed with five MSSA and five MRSA isolates using the broth microdilution and chequerboard assays, respectively. The *in vivo* efficacy of cefazolin plus fosfomycin for the treatment of MRSA infections was assessed using the *Galleria mellonella* survival assay.

**Results:** Using fractional inhibitory concentration index (FICI), the evaluated combination of cefazolin plus fosfomycin showed synergistic *in vitro* activity against all MSSA and MRSA isolates tested. In addition, cefazolin susceptibility was recovered in all MRSA isolates except one fosfomycin-resistant strain when combined with fosfomycin at readily achievable concentrations. The *G. mellonella* survival assay demonstrated highly synergistic *in vivo* activity of cefazolin plus fosfomycin, resulting in a 44–52% reduction in mortality when compared to cefazolin-alone and fosfomycin-alone, respectively.

**Conclusion:** If susceptibility to fosfomycin is either confirmed or can be assumed based on local resistance patterns, combination therapy with cefazolin plus fosfomycin could be a valuable treatment option for empirical as well as targeted therapy of *S. aureus* and MRSA infections. Future studies proving the clinical significance of this combination therapy are therefore warranted.

## Introduction

Over recent years, fosfomycin has raised considerable interest due to its potent activity against a wide spectrum of problematic pathogens including *Staphylococcus aureus*, the leading cause of bacteremia and infective endocarditis (IE). According to current guidelines vancomycin or daptomycin are recommended for treatment of bacteremia and IE caused by methicillin-resistant *Staphylococcus aureus* (MRSA), although bactericidal activity of glycopeptides is poorer than that of beta-lactams and their penetration into endocardial vegetations is markedly lower (Stevens, 2006; Habib et al., 2015). Daptomycin shows rapid bactericidal activity and is therefore a reliable alternative for severe MRSA infections (Richter et al., 2003; Habib et al., 2015). However, it possesses some shortcomings, including a strong inoculum effect and notable rates of emergent resistance in patients with left-sided IE, highlighting the need for alternative regimens and rescue therapies (Fowler et al., 2006; Moise et al., 2009; Morrisette et al., 2020). Due to its broad antimicrobial activity against Gram-positive and Gram-negative organisms, fosfomycin has been studied in combination with various beta-lactam antibiotics because of their wide therapeutic range and strong clinical efficacy. These combinations have shown highly synergistic activity against MRSA, especially when fosfomycin was studied together with imipenem (Grif et al., 2001; del Río et al., 2016). A multicenter clinical trial investigating the efficacy and safety of fosfomycin plus imipenem as rescue therapy for complicated MRSA bacteremia and IE showed that this combination therapy is a safe and effective alternative and should be further investigated (del Río et al., 2014). However, given the global public-healthcare issue posed by the emergence and rapid spread of carbapenem resistance, the restrained use of carbapenems, especially for treatment of Gram-positive infections for which reliable alternatives are still available, is of utmost importance (Meletis, 2016). Therefore, we selected the narrow-spectrum beta-lactam cefazolin for combination with fosfomycin, which has shown good clinical efficacy and tolerability in the treatment of MSSA infections (Loubet et al., 2018).

## Methods

### Bacterial Strains

Ten *S. aureus* isolates were tested in this study: five methicillin- and fosfomycin-susceptible (ATCC-29213 and four clinical isolates), one methicillin- and fosfomycin-resistant (DSMZ-23622) and four methicillin-resistant and fosfomycin-susceptible (ATCC-33592 and three clinical isolates) isolates. All clinical isolates were routinely obtained from positive blood cultures and identified by routine microbiological methods including Matrix-Assisted Laser Desorption/ionization Time-Of-Flight Mass Spectrometry (MALDI-TOF MS, MALDI Biotyper smart with the Compass IVD software v4.2, Bruker Daltonics GmbH, Germany) (Supplementary Table S1). In addition, all isolates were tested by polymerase chain reaction (PCR) for the presence of the methicillin-resistance gene *mecA* as previously described by Terpstra *et al.* (Terpstra et al., 1999).

### Antimicrobial Susceptibility and Synergy Testing

Minimum inhibitory concentrations (MICs) for cefazolin and fosfomycin were determined by broth microdilution method in cation-adjusted Mueller-Hinton broth (CA-MHB) supplemented with glucose-6-phosphate (G6P) at a final concentration of 25 mg/L which was also used for synergy testing.

Synergy-testing was performed using a chequerboard assay as previously described (Li et al., 2018). Briefly, serial dilutions of cefazolin and fosfomycin were made in u-bottomed 96-well microtiter plates with a final inoculum of approximately 5 × 10^5^ CFU/ml and a final volume of 200 µL per well. Plates were read after an incubation of 18–24 h at 36°C (±1°C). After calculation of the fractional inhibitory concentration indices (FICI) results were interpreted as synergism ≤0.5, >0.5–4 = no interaction and >4 antagonism. The susceptible breakpoint index (SBPI) was calculated according to the following formula: SBPI = (susceptible breakpoint of antimicrobial A/combined MIC of antimicrobial A) + (susceptible breakpoint of antimicrobial B/combined MIC of antimicrobial B), using the clinical breakpoint of 32 mg/L for fosfomycin and the pharmacokinetic/pharmacodynamic breakpoint of 2 mg/L for cefazolin (Milne and Gould, 2010; The European Committee on Antimicrobial Susceptibility Testing, 2021. Breakpoint tables for interpretation of MICs and zone diameters. Version 11.0, 2021. http://www.eucast.org). An SBPI ≥2 indicates that the combined MICs of the tested antimicrobials are equally or lower than their respective breakpoints. It follows that the greater the SBPI value, the more effective the antimicrobial combination is. All experiments were performed in duplicates.

### Penicillin-Binding Protein Expression Analysis

Relative gene expression of penicillin-binding protein 1 (PBP1), PBP2, PBP2′ (also called PBP2a), PBP3, and PBP4 was determined for one fosfomycin-susceptible (ATCC-33592) and one fosfomycin-resistant (DSMZ-23622) MRSA after a 4 h incubation with either fosfomycin or cefazolin at 0.25xMIC or without antibiotics as control. Bacterial inocula were prepared by diluting overnight cultures with fresh tryptic soy broth (TSB) followed by an incubation period on an orbital shaker at 36°C (±1°C) to achieve exponential growth. RNA was extracted using lysing matrix tubes (MP Biomedicals) and the FavorPrep-Tissue Total RNA Mini-Kit (Favorgen Biotech Corp, Taiwan). Copy-DNA was obtained using the Onescript cDNA Synthesis-Kit (ABMgood, Canada) and RT-PCR was performed with low-ROX BrightGreen qPCR Mastermix (ABMgood, Canada) using previously described primers for PBPs and *gap*, which encodes for the glyceraldehyde-3-phosphate dehydrogenase, as housekeeping gene (Supplementary Table S2) (Navratna et al., 2010). All experiments were performed in quadruplicates and relative expression values (±SD) were calculated by ΔΔCt using no treatment controls as references.

### *In vivo Galleria mellonella* Survival Assay

A fosfomycin-susceptible MRSA (ATCC-33592) was used for the *in vivo G. mellonella* survival assay. Bacterial inocula were prepared by diluting overnight cultures with fresh TSB followed by incubation of 4 h on an orbital shaker at 36°C (±1°C) to obtain bacteria in exponential growth phase and with a cell density causing a mortality rate of ≥80% within 5 days post infection. *G. mellonella* larvae were originally obtained from TruLarv^™^ (Biosystems Technology), further bred in our laboratory and used at a weight between 220 and 280 mg, after a 24-h fasting period. After random distribution into four treatment groups: infected control, cefazolin-alone, fosfomycin-alone or cefazolin plus fosfomycin, infection of the larvae was performed by injecting 10 µL (∼7 × 10^8^ CFU/ml) of the bacterial inoculum into one of the last prolegs using a 50 µL Hamilton syringe (Merck, Darmstadt, Germany). One hour after infection, a single dose of antibiotics was administered into another proleg to minimize leakage of the hemolymph. For cefazolin the human dose of 100 mg/kg was used whereas the fosfomycin dose (0.8 mg/kg) was determined in preliminary experiments to achieve mortality rates of 60–90% (Supplementary Figure S1). For the entire experiment, larvae were incubated at 37°C for five days and survival was measured every 24 h. The first experiment contained 20–25 larvae per treatment group, while the duplicate experiment was performed with 10–15 larvae from a different batch on a different day (n per treatment group = 36–40). Both infected as well as uninfected larvae, which only received sterile PBS, served as controls. In addition, drug toxicity was ruled out by tracking the survival of 10 larvae each after a single dose of 200 mg/kg fosfomycin and 100 mg/kg cefazolin. Survival curves were plotted using GraphPad Prism v6.01 (GraphPad Software Inc. San Diego) and analyzed using the log-rank test.

## Results

### Antimicrobial Susceptibility and Synergy Testing

Five of the ten *S. aureus* isolates tested positive for the presence of *mecA*, namely ATCC-33592.

DSMZ-23622, 874/19, 845/19, 563/18 (Supplementary Figure S2).

The results of the *in vitro* susceptibility and synergy testing are summarized in Table 1. All isolates showed a FICI ≤0.5 for the combination of cefazolin plus fosfomycin indicating synergism. In addition, all isolates except the fosfomycin-resistant MRSA demonstrated a SBPI >2.

**TABLE 1:** Summary of *in vitro* susceptibility and synergy testing.

Isolates	MIC (mg/L)		FICI-interpretation^a^ (Mean-FICI ±SD	Combined MICs (CEF; FOF)^b^	Recovered CEF susceptibility (min Combined FOF-MIC)^c^	SBPI^d^
	CEF	FOF				
MSSA						
ATCC-29213	0.5	2	Sy (0.28 ± 0.04)	1/4; 1/16	n.a	288
280/20	0.25	2	Sy (0.41 ± 0.13)	1/8; 1/4	n.a	144
249/20	0.25	0.5	Sy (0.5 ± 0.00)	1/4; 1/4	n.a	288
204/20	0.5	2	Sy (0.34 ± 0.04)	1/16; 1/4	n.a	112
231/20	0.5	2	Sy (0.25 ± 0.00)	1/8; 1/8	n.a	160
MRSA						
ATCC-33592	128	8	Sy (0.14 ± 0.02)	1/32; 1/16	S (≥1/8)	64.4
DSMZ-23622	1,024	128	Sy (0.31 ± 0.00)	1/16; 1/4	R	1.1
874/19	128	8	Sy (0.13 ± 0.00)	1/512; 1/8	S (≥1/8)	40
845/19	64	1	Sy (0.05 ± 0.01)	1/64; 1/32	S (≥1/32)	1,026
563/18	64	2	Sy (0.04 ± 0.01)	1/128; 1/32	S (≥1/64)	516

*MIC, minimum inhibitory concentrations; CEF, cefazolin; FOF, fosfomycin; FICI, fractional inhibitory concentration index; SBPI, susceptible breakpoint index; MSSA, methicillin-susceptible *Staphylococcus aureus*; MRSA methicillin-resistant *Staphylococcus aureus*; ATCC, American type culture collection; DSMZ, german collection of microorganisms and cell cultures.

a Interpretation of the FICI: Sy, synergism = ≤0.5, AE, additive effect = 0.5–≤1.0, NI, no interaction = >1 and <4; An, antagonism = ≥4, followed by the mean FICI ±SD in brackets.

b Combined minimum inhibitory concentrations of cefazolin and fosfomycin used for calculation of the fractional inhibitory concentration index stated as relative concentrations of their respective MICs.

c Stated as susceptible (S) when the lowest combined cefazolin concentration, obtained at fosfomycin concentrations below its susceptible breakpoint of 32 mg/L, was below its pharmacokinetic/pharmacodynamic breakpoint of 2 mg/L. The lowest, respective fosfomycin concentrations which resulted in susceptible cefazolin MICs are stated as times of their MIC. N. a. not applicable due to the cefazolin MIC below the *p*K/PD breakpoint.

d For calculation of the SBPI the clinical breakpoint of 32 mg/L for intravenous fosfomycin and the pharmacokinetic/pharmacodynamic breakpoint of 2 mg/L for cefazolin were used, both obtained from the European Committee on Antimicrobial Susceptibility Testing.

### *In vivo G.mellonella* Survival Assay

**FIGURE 1:**
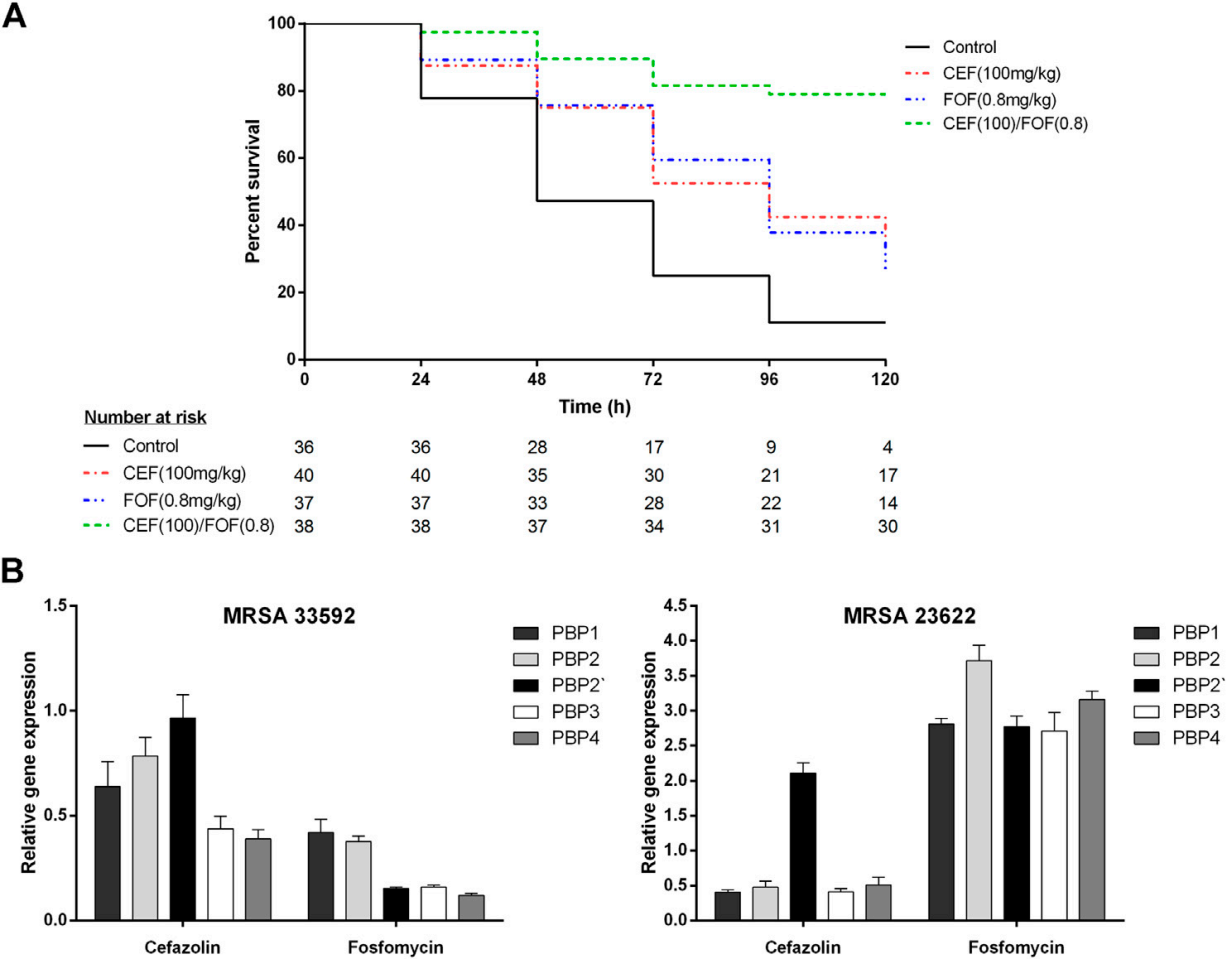
**(A)** Survival curves of *G. mellonella* larvae infected with methicillin-resistant *Staphylococcus aureus* (ATCC-33592) followed by treatment with cefazolin (100 mg/kg), fosfomycin (0.8 mg/kg) or the combination of both (cefazolin 100 mg/kg plus fosfomycin 0.8 mg/kg). Curves represent the pooled data of two experiments performed on separate days. **(B)** Relative gene expression of penicillin binding proteins (PBP1, PBP2, PBP2′, PBP3, and PBP4) determined for one fosfomycin-susceptible (ATCC-33592) and one fosfomycin-resistant (DSMZ-23622) methicillin-resistant *Staphylococcus aureus* (MRSA) isolate using RT-PCR with gap as housekeeping gene and a no treatment control as reference. Bacteria were exposed to either cefazolin or fosfomycin at a concentration corresponding to 0.25 times of their respective minimum inhibitory concentrations for a time period of 4 h. Data is stated as mean (±SD) relative quantification values. *CEF, cefazolin; FOF, fosfomycin; MRSA, methicillin-resistant *Staphylococcus aureus*; PBP, penicillin-binding protein.

The control group infected with MRSA (ATCC-33592) showed a mortality of 89% within 5 days. Fosfomycin-alone (200 mg/kg) was highly effective and resulted in a survival rate of 100% (Supplementary Figure S1), whereas cefazolin-alone (100 mg/kg) resulted in a mortality rate of 65%. When cefazolin was combined with low-dose fosfomycin (0.8 mg/kg), which achieved 73% mortality alone, mortality decreased to 21% (*p* = 0.0002 for combination vs. cefazolin-alone; *p* < 0.0001 for combination vs fosfomycin-alone), as shown in Figure 1A.

### Penicillin-Binding Protein Expression Analysis

Data of the PBP expression analysis is demonstrated in Figure 1B; Supplementary Table S3. After exposure to cefazolin (0.25xMIC), both MRSA showed reduced expression of PBP1, PBP2, PBP3 and PBP4, while PBP2′ remained unchanged in the fosfomycin-susceptible MRSA (ATCC-33592) and was even overexpressed in the fosfomycin-resistant isolate (DSMZ-23622). Exposure to fosfomycin (0.25xMIC) reduced the expression of all PBPs and most significantly PBP2′, PBP3, and PBP4 in the fosfomycin-susceptible isolate while overexpression of all PBPs was observed for the fosfomycin-resistant strain.

## Discussion

This study demonstrated the highly synergistic activity of cefazolin plus fosfomycin against both, MSSA and MRSA. With regard to the FICI, all isolates showed synergistic activity, which was even more pronounced in fosfomycin-susceptible MRSA. When combined with fosfomycin at readily achievable concentrations, all of these isolates regained susceptibility to cefazolin and demonstrated SBPIs ≥40. Furthermore, cefazolin susceptibility of an MRSA was recovered *in vivo* by combination with low-dose fosfomycin, resulting in significantly reduced mortality of at least 44% (Figure 1A). In contrast, the fosfomycin-resistant MRSA showed synergistic activity with respect to the FICI but failed to recover its susceptibility to cefazolin and consequently achieved only an SBPI of 1.1, highlighting the importance of additional parameters to evaluate *in vitro* synergies regarding their potential clinical relevance (Table 1) (Milne and Gould, 2010). The data obtained in the present study are consistent with a previous study by Grif *et al.* that demonstrated synergistic *in vitro* activity of fosfomycin plus cefazolin and fosfomycin plus meropenem against five *S. aureus* strains, including a glycopeptide-intermediate *S. aureus* and an MRSA (Grif et al., 2001). However, in this previous contribution to the field no fosfomycin-resistant isolate was tested and neither individual nor combination MICs of the antimicrobials tested are reported, so the extent of synergistic activity cannot be compared (Grif et al., 2001).

The differences between the fosfomycin-susceptible and resistant MRSA isolates were also observed in PBP expression analysis when both strains were exposed to fosfomycin. The fosfomycin-susceptible strain showed an overall reduction in PBP expression with a shift toward PBP1 and PBP2 whereas the fosfomycin-resistant strain overexpressed all PBPs with a slight shift toward PBP2 (Figure 1B). This is consistent with the overall reduction of PBPs, including PBP2′, determined fluorographically by Utsui *et al.* In a more recent study only a reduction of PBP1 and PBP2, but not PBP2′, was observed using SDS-PAGE electrophoresis, although fosfomycin-susceptible MRSA were studied at comparable concentrations (Utsui et al., 1986; del Río et al., 2016). Thus, given the reduced but still synergistic activity of cefazolin plus fosfomycin, observed in the present study in a fosfomycin-resistant MRSA without a shift or reduction in PBP expression, the previously hypothesized mechanisms of synergy can only partially explain these findings (Utsui et al., 1986; Najioullah et al., 1992; del Río et al., 2016).

Despite efforts to optimize management, MRSA bloodstream infections still demonstrate high mortality rates >30% regardless of the antimicrobial therapy used (Gasch et al., 2013; Veganzones et al., 2019). In addition, the increasing prevalence of MRSA worldwide was associated to epidemiological changes such as older age and the increased presence of comorbidities, highlighting the need for safe and effective treatment alternatives (Gasch et al., 2013; Veganzones et al., 2019). In light of these facts, del Rio *et al.* investigated imipenem plus fosfomycin for treatment of MRSA bacteremia and IE after failure of vancomycin or daptomycin, demonstrating its safety and efficacy (del Río et al., 2014).

However, based on the data obtained in the present study, cefazolin plus fosfomycin may represent an effective therapeutic option for MRSA and MSSA infections, including settings of unknown beta-lactam susceptibility and may help to maintain carbapenems as reliable last-resort treatment. Thus, further studies proving its clinical significance are warranted.

### Transparency Declaration

Dr Vossen reports personal fees from Astro Pharma, other from Infectopharm, outside the submitted work.

## Data Availability

The original contributions presented in the study are included in the article/Supplementary Material, further inquiries can be directed to the corresponding authors.

## References

[B1] del Río A. García-de-la-Mària C. Entenza J. M. Gasch O. Armero Y. Soy D. (2016). Fosfomycin Plus β-Lactams as Synergistic Bactericidal Combinations for Experimental Endocarditis Due to Methicillin-Resistant and Glycopeptide-Intermediate *Staphylococcus aureus* . Antimicrob. Agents Chemother. 60, 478–486. 10.1128/AAC.02139-15 26525803 PMC4704234

[B2] del Río A. Gasch O. Moreno A. Peña C. Cuquet J. Soy D. (2014). Efficacy and Safety of Fosfomycin Plus Imipenem as Rescue Therapy for Complicated Bacteremia and Endocarditis Due to Methicillin-Resistant *Staphylococcus aureus*: A Multicenter Clinical Trial. Clin. Infect. Dis. 59, 1105–1112. 10.1093/cid/ciu580 25048851

[B3] Fowler V. G. Boucher H. W. Corey G. R. Abrutyn E. Karchmer A. W. Rupp M. E. (2006). Daptomycin versus Standard Therapy for Bacteremia and Endocarditis Caused byStaphylococcus Aureus. N. Engl. J. Med. 355, 653–665. 10.1056/NEJMoa053783 16914701

[B4] Gasch O. Camoez M. Dominguez M. A. Padilla B. Pintado V. Almirante B. (2013). Predictive Factors for Mortality in Patients with Methicillin-Resistant *Staphylococcus aureus* Bloodstream Infection: Impact on Outcome of Host, Microorganism and Therapy. Clin. Microbiol. Infect. 19, 1049–1057. 10.1111/1469-0691.12108 23331461

[B5] Grif K. Dierich M. P. Pfaller K. Miglioli P. A. Allerberger F. (2001). *In vitro* activity of Fosfomycin in Combination with Various Antistaphylococcal Substances. J. Antimicrob. Chemother. 48, 209–217. 10.1093/jac/48.2.209 11481290

[B6] Habib G. Lancellotti P. Antunes M. J. Bongiorni M. G. Casalta J.-P. Del Zotti F. (2015). 2015 ESC Guidelines for the Management of Infective Endocarditis. Eur. Heart J. 36, 3075–3128. 10.1093/eurheartj/ehv319 26320109

[B7] Li L. Chen H. Liu Y. Xu S. Wu M. Liu Z. (2020). Synergistic Effect of Linezolid with Fosfomycin against *Staphylococcus aureus* In Vitro and in an Experimental *Galleria* Mellonella Model. J. Microbiol. Immunol. Infect. 53, 731–738. 10.1016/j.jmii.2018.12.007 30638785

[B8] Loubet P. Burdet C. Vindrios W. Grall N. Wolff M. Yazdanpanah Y. (2018). Cefazolin versus Anti-staphylococcal Penicillins for Treatment of Methicillin-Susceptible *Staphylococcus aureus* Bacteraemia: a Narrative Review. Clin. Microbiol. Infect. 24, 125–132. 10.1016/j.cmi.2017.07.003 28698037

[B9] Meletis G. (2016). Carbapenem Resistance: Overview of the Problem and Future Perspectives. Ther. Adv. Infect. 3, 15–21. 10.1177/2049936115621709 PMC4735501 26862399

[B10] Milne K. E. N. Gould I. M. (2010). Combination Testing of Multidrug-Resistant Cystic Fibrosis Isolates of *Pseudomonas aeruginosa*: Use of a New Parameter, the Susceptible Breakpoint Index. J. Antimicrob. Chemother. 65, 82–90. 10.1093/jac/dkp384 19861334

[B11] Moise P. A. North D. Steenbergen J. N. Sakoulas G. (2009). Susceptibility Relationship between Vancomycin and Daptomycin in *Staphylococcus aureus*: Facts and Assumptions. Lancet Infect. Dis. 9, 617–624. 10.1016/S1473-3099(09)70200-2 19778764

[B12] Morrisette T. Alosaimy S. Abdul-Mutakabbir J. C. Kebriaei R. Rybak M. J. (2020). The Evolving Reduction of Vancomycin and Daptomycin Susceptibility in MRSA-Salvaging the Gold Standards with Combination Therapy. Antibiotics 9, 762. 10.3390/antibiotics9110762 PMC7692208 33143290

[B13] Najioullah F. Pellon G. r. Freney J. Michel G. Fleurette J. (1992). Fosfomycin Enhances the Expression of Penicillin-Binding Protein 2 in Methicillin-Sensitive and Methicillin-resistantStaphylococcus Aureusstrains. FEMS Microbiol. Lett. 97, 221–226. 10.1111/j.1574-6968.1992.tb05467.x 1427011

[B14] Navratna V. Nadig S. Sood V. Prasad K. Arakere G. Gopal B. (2010). Molecular Basis for the Role of *Staphylococcus aureus* Penicillin Binding Protein 4 in Antimicrobial Resistance. Jb 192, 134–144. 10.1128/JB.00822-09 PMC2798245 19854906

[B15] Richter S. S. Kealey D. E. Murray C. T. Heilmann K. P. Coffman S. L. Doern G. V. (2003). The In Vitro Activity of Daptomycin against *Staphylococcus aureus* and *Enterococcus* Species. J. Antimicrob. Chemother. 52, 123–127. 10.1093/jac/dkg288 12805265

[B16] Stevens D. L. (2006). The Role of Vancomycin in the Treatment Paradigm. Clin. Infect. Dis. 42 (Suppl. 1), S51–S57. 10.1086/491714 16323121

[B17] Terpstra S. Noordhoek G. T. Voesten H. G. J. Hendriks B. Degener J. E. (1999). Rapid Emergence of Resistant Coagulase-Negative Staphylococci on the Skin after Antibiotic Prophylaxis. J. Hosp. Infect. 43, 195–202. 10.1053/JHIN.1999.0636 10582186

[B18] The European Committee on Antimicrobial Susceptibility Testing (2021). Breakpoint Tables for Interpretation of MICs and Zone Diameters. Version 11.0. http://www.eucast.org.

[B19] Utsui Y. Ohya S. Magaribuchi T. Tajima M. Yokota T. (1986). Antibacterial Activity of Cefmetazole Alone and in Combination with Fosfomycin against Methicillin- and Cephem-Resistant *Staphylococcus aureus* . Antimicrob. Agents Chemother. 30, 917–922. 10.1128/aac.30.6.917 3468883 PMC180619

[B20] Veganzones J. Montero A. Maseda E. (2019). New Evidence on the Use of Fosfomycin for Bacteremia and Infectious Endocarditis. Rev. Esp Quimioter 32, 25–29. 31131589 PMC6555165

