# Supplementary material for: Synergistic Effect of Cefazolin Plus Fosfomycin Against Staphylococcus aureus in vitro and in vivo in an Experimental Galleria mellonella Model
Source: Front Pharmacol. 2021 May 11;12:685807. doi: 10.3389/fphar.2021.685807 (PMC8144499; doi:10.3389/fphar.2021.685807)

**Supplementary Table S1.** Results of bacterial identification by Matrix-Assisted Laser Desorption/Ionization Time-Of-Flight Mass Spectrometry (MALDI-TOF MS) shown as matched pattern, score value, and NCBI identification number of best match.

| **Isolates** |  | **MALDI-TOF MS**  **Score Value** | | |
| --- | --- | --- | --- | --- |
|  |  | **Matched Pattern** | **Score Value** | **NCBI Identifier** |
| ***MSSA*** |  |  |  |  |
| ATCC-29213 |  | Staphylococcus aureus ssp aureus DSM 3463 DSM | 2.20 | 46170 |
| 280/20 |  | Staphylococcus aureus ssp aureus DSM 3463 DSM | 2.16 | 46170 |
| 249/20 |  | Staphylococcus aureus ssp aureus DSM 3463 DSM | 2.12 | 46170 |
| 204/20 |  | Staphylococcus aureus ATCC 33862 THL | 2.15 | 1280 |
| 231/20 |  | Staphylococcus aureus ATCC 33591 THL | 2.12 | 1280 |
|  |  |  |  |  |
| ***MRSA*** |  |  |  |  |
| ATCC-33592 |  | Staphylococcus aureus ssp aureus DSM 3463 DSM | 2.12 | 46170 |
| DSMZ-23622 |  | Staphylococcus aureus ssp aureus DSM 3463 DSM | 2.19 | 46170 |
| 874/19 |  | Staphylococcus aureus ATCC 33591 THL | 2.04 | 1280 |
| 845/19 |  | Staphylococcus aureus ATCC 33591 THL | 2.12 | 1280 |
| 563/18 |  | Staphylococcus aureus ssp aureus DSM 799 DSM | 2.28 | 46170 |

* MSSA, methicillin-susceptible Staphylococcus aureus; MRSA methicillin-resistant Staphylococcus aureus; ATCC, American type culture collection; DSMZ, german collection of microorganisms and cell cultures; MALDI-TOF MS, Matrix-Assisted Laser Desorption/Ionization Time-Of-Flight Mass Spectrometry; NCBI, National Center for Biotechnology Information

**Supplementary Table S2.** Oligonucleotides used for PCR analysis

| **Gene** | **Forward oligonucleotide (5’ – 3’)** | **Reverse oligonucleotide (5’ – 3’)** | **References** |
| --- | --- | --- | --- |
| **PBP1** | 5‘-ACCAATAACAAAAGATACTGCTGAA-3‘ | 5‘-CGTCACCCATAAAACTTACAAAATA-3‘ | [1] |
| **PBP2** | 5‘-TAACAGAAACCAAGCAACAGAT-3‘ | 5‘-TTTGTCGTAAAGCATCATAAATAGA-3‘ | [1] |
| **PBP2a** | 5‘-CAACTAATGAAACAGAAAGTCGTAA-3‘ | 5‘-TAATGTATGTGCGATTGTATTGCTA-3‘ | [1] |
| **PBP3** | 5‘-ACAAAATCCTAAAAATGGAGACA-3‘ | 5‘-ACCTTGGAAATGTAATGGTTCAT-3‘ | [1] |
| **PBP4** | 5‘-ACGATGTTTTACCAAGTGATTTTAG-3‘ | 5‘-ACCAATGATAGTGAATAATGGATGT-3‘ | [1] |
| **gap** | 5‘-CAAAATACACAAGACGCACC-3‘ | 5‘-CCGATAGCTTTAGCAGCAC-3‘ | [*] |
| **mecA** | 5′-GTTGTAGTTGTCGGGTTTGG-3’ | 5′-CTTCCACATACCATCTTCTTTAAC-3’ | [2] |

PBP1-4, penicillin binding protein 1-4; gap, the Staphylococcus aureus gene encoding the glyceraldehyde-3-phosphate dehydrogenase

[1] Navratna V, Nadig S, Sood V, Prasad K, Arakere G, Gopal B. Molecular basis for the role of Staphylococcus aureus penicillin binding protein 4 in antimicrobial resistance. *J Bacteriol* 2010; **192**: 134–44.

[2] Terpstra S, Noordhoek GT, Voesten HG, Hendriks B, Degener J. Rapid emergence of resistant coagulase-negative staphylococci on the skin after antibiotic prophylaxis. The Journal of hospital infection. 1999

[*] These primers were designed for the present study

**Supplementary Table S3.** Relative gene expression of penicillin binding proteins (PBP1, PBP2, PBP2`, PBP3 and PBP4) determined for one fosfomycin-susceptible (ATCC-33592) and one fosfomycin-resistant (DSMZ-23622) MRSA isolate using RT-PCR with gap as housekeeping gene and a no treatment control as reference. Bacteria were exposed to either cefazolin or fosfomycin at a concentration corresponding to 0.25 times of their respective minimum inhibitory concentrations (MIC) for a time period of 4 hours. Data is stated as mean (±SD) relative quantification values and individual Ct values

|  |  | **MRSA 33592** | | **MRSA 23622** | |
| --- | --- | --- | --- | --- | --- |
|  |  | **Mean RQ (±SD)** | **Ct values** | **Mean RQ (±SD)** | **Ct values** |
| **NTC** | **pbp1** | 1.000 (±0.120) | \| 25,419 \| \| --- \| \| 25,465 \| \| 25,417 \| \| 25,238 \| | 1.000 (±0.080) | \| 27,619 \| \| --- \| \| 27,753 \| \| 27,723 \| \| 27,720 \| |
|  |  |  |  |  |  |
| **CEFA 0.25xMIC** | **pbp1** | 0.639 (±0.119) | \| 26,827 \| \| --- \| \| 26,738 \| \| 27,304 \| \| 26,971 \| | 0.405 (±0.034) | \| 28,021 \| \| --- \| \| 28,212 \| \| 28,116 \| |
|  |  |  |  |  |  |
| **FOF 0.25xMIC** | **pbp1** | 0.420 (±0.061) | \| 26,149 \| \| --- \| \| 26,284 \| \| 26,618 \| \| 26,147 \| | 2.807 (±0.079) | \| 24,939 \| \| --- \| \| 24,966 \| \| 24,960 \| \| 25,001 \| |
|  |  |  |  |  |  |
| **NTC** | **pbp2** | 1.000 (±0.129) | \| 25,328 \| \| --- \| \| 25,179 \| \| 25,476 \| \| 25,265 \| | 1.000 (±0.145) | \| 28,332 \| \| --- \| \| 27,937 \| \| 28,079 \| \| 27,887 \| |
|  |  |  |  |  |  |
| **CEFA 0.25xMIC** | **pbp2** | 0.784 (±0.089) | \| 26,615 \| \| --- \| \| 26,580 \| \| 26,654 \| \| 26,519 \| | 0.476 (±0.085) | \| 28,484 \| \| --- \| \| 28,452 \| \| 28,080 \| \| 27,937 \| |
|  |  |  |  |  |  |
| **FOF 0.25 xMIC** | **pbp2** | 0.376 (±0.027) | \| 26,483 \| \| --- \| \| 26,430 \| \| 26,259 \| \| 26,385 \| | 3.718 (±0.219) | \| 24,967 \| \| --- \| \| 24,908 \| \| 24,985 \| \| 24,804 \| |
|  |  |  |  |  |  |
| **NTC** | **pbp2a** | 1.000 (±0.110) | \| 24,203 \| \| --- \| \| 24,258 \| \| 24,117 \| \| 24,147 \| | 1.000 (±0.102) | \| 27,127 \| \| --- \| \| 27,263 \| \| 27,034 \| |
|  |  |  |  |  |  |
| **CEFA 0.25xMIC** | **pbp2a** | 0.966 (±0.112) | \| 25,204 \| \| --- \| \| 25,134 \| \| 25,077 \| \| 25,227 \| | 2.109 (±0.144) | \| 25,205 \| \| --- \| \| 25,141 \| \| 25,234 \| \| 25,113 \| |
|  |  |  |  |  |  |
| **FOF 0.25xMIC** | **pbp2a** | 0.151 (±0.009) | \| 26,536 \| \| --- \| \| 26,518 \| \| 26,681 \| \| 26,541 \| | 2.773 (±0.151) | \| 24,359 \| \| --- \| \| 24,496 \| \| 24,475 \| \| 24,356 \| |
|  |  |  |  |  |  |
| **NTC** | **pbp3** | 1.000 (±0.128) | \| 24,964 \| \| --- \| \| 24,844 \| \| 24,974 \| \| 24,716 \| | 1.000 (±0.131) | \| 27,718 \| \| --- \| \| 27,571 \| \| 27,509 \| \| 27,299 \| |
|  |  |  |  |  |  |
| **CEFA 0.25xMIC** | **pbp3** | 0.437 (±0.061) | \| 26,964 \| \| --- \| \| 27,103 \| \| 26,813 \| \| 27,112 \| | 0.409 (±0.048) | \| 27,775 \| \| --- \| \| 28,086 \| \| 27,803 \| \| 28,032 \| |
|  |  |  |  |  |  |
| **FOF 0.25xMIC** | **pbp3** | 0.159 (±0.009) | \| 27,171 \| \| --- \| \| 27,278 \| \| 27,138 \| | 2.711 (±0.262) | \| 24,964 \| \| --- \| \| 24,953 \| \| 24,751 \| \| 24,680 \| |
|  |  |  |  |  |  |
| **NTC** | **pbp4** | 1.000 (±0.191) | \| 25,720 \| \| --- \| \| 25,471 \| \| 25,195 \| | 1.000 (±0.077) | \| 27,894 \| \| --- \| \| 27,904 \| \| 27,993 \| \| 27,976 \| |
|  |  |  |  |  |  |
| **CEFA 0.25xMIC** | **pbp4** | 0.389 (±0.043) | \| 27,725 \| \| --- \| \| 27,786 \| \| 27,747 \| | 0.507 (±0.113) | \| 27,810 \| \| --- \| \| 27,935 \| \| 28,556 \| \| 27,821 \| |
|  |  |  |  |  |  |
| **FOF 0.25xMIC** | **pbp4** | 0.119 (±0.010) | \| 28,295 \| \| --- \| \| 28,142 \| \| 28,278 \| \| 28,058 \| | 3.160 (±0.123) | \| 25,000 \| \| --- \| \| 25,083 \| \| 25,064 \| \| 24,986 \| |
|  |  |  |  |  |  |
| **NTC** | **gap** |  | \| 21,708 \| \| --- \| \| 21,767 \| \| 21,415 \| \| 21,588 \| |  | \| 26,132 \| \| --- \| \| 26,174 \| \| 25,970 \| \| 25,977 \| |
|  |  |  |  |  |  |
| **CEFA 0.25xMIC** | **gap** |  | \| 22,560 \| \| --- \| \| 22,632 \| \| 22,688 \| \| 22,315 \| |  | \| 25,268 \| \| --- \| \| 25,139 \| \| 25,107 \| |
|  |  |  |  |  |  |
| **FOF 0.25xMIC** | **gap** |  | \| 21,342 \| \| --- \| \| 21,250 \| \| 21,309 \| \| 21,237 \| |  | \| 24,831 \| \| --- \| \| 24,813 \| \| 24,771 \| \| 24,844 \| |

*MRSA, methicillin-resistant Staphylococcus aureus; mean RQ, mean relative quantification values (±standard deviation) calculated by ΔΔCt using a no treatment control as reference; NTC, no treatment control; pbp, penicillin binding protein; CEF, cefazolin; FOF, fosfomycin; MIC, minimum inhibitory concentration: gap, the Staphylococcus aureus gene encoding the glyceraldehyde-3-phosphate dehydrogenase



**Supplementary Figure S1.** Survival curves of *G.mellonella* larvae infected with methicillin-resistant *Staphylococcus aureus* (ATCC-33592) followed by treatment with fosfomycin at doses of 200mg/kg (20 larvae), 50mg/kg (19 larvae), 25mg/kg (19 larvae), 12.5mg/kg (20 larvae), 6.25mg/kg (20 larvae) and 0.1mg/kg (12 larvae). The control curve represents the pooled data of three experiments performed on separate days (32 larvae, 11 larvae, 20 larvae). Data sets of fosfomycin 200mg/kg, 50mg/kg, 25mg/kg and 12.5mg/kg were nudged to prevent an overlap.

**Supplementary Figure S2.** Agarose gel electrophoresis for the detection of the mecA resistance gene. 1: molecular weight marker (100bp Plus Opti DNA Marker, ABMGood, Canada); 2. 280/20; 3. 249/20; 4. 204/20; 5. 231/20; 6. 874/19; 7. 845/19; 8. 563/18; 9. ATCC-29213 (internationally standardized methicillin-susceptible Staphylococcus aureus strain); 10. ATCC-33592 (internationally standardized methicillin-resistant Staphylococcus aureus strain); 11. DSMZ-23612; 12. molecular weight marker (100bp Plus Opti DNA Marker, ABMGood, Canada); 13. negative control
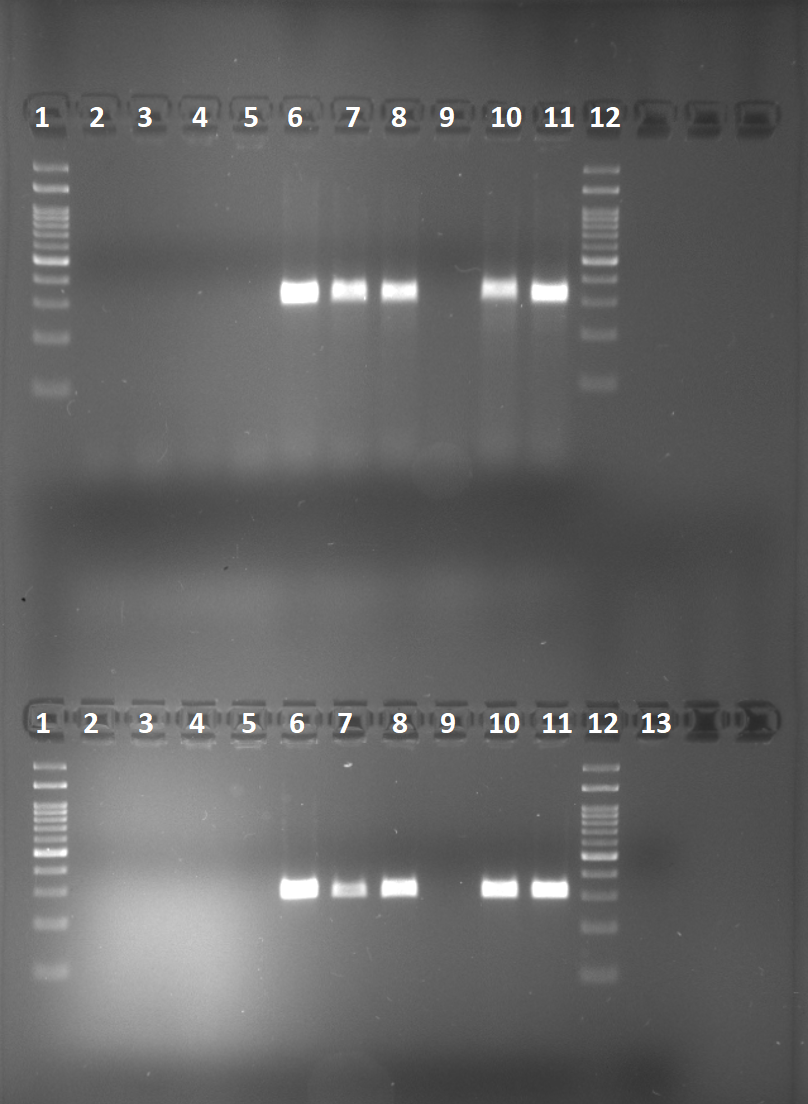

Supplement: Supplementary file 1 [file DataSheet1.docx]
